# Metastatic Granulosa Cell Tumor of the Testis: Clinical Presentation and Management

**DOI:** 10.1155/2016/9016728

**Published:** 2016-05-15

**Authors:** Anand Mohapatra, Aaron M. Potretzke, Brent A. Knight, Min Han, Robert S. Figenshau

**Affiliations:** ^1^Division of Urologic Surgery, Washington University School of Medicine, St. Louis, MO 63110, USA; ^2^Department of Pathology, Washington University School of Medicine, St. Louis, MO 63110, USA

## Abstract

Granulosa cell tumors (GCTs) of the testis are rare sex cord-stromal tumors that are present in both juvenile and adult subtypes. While most adult GCTs are benign, those that present with distant metastases manifest a grave prognosis. Treatments for aggressive GCTs are not well established. Options that have been employed in previous cases include retroperitoneal lymph node dissection (RPLND), radiation, chemotherapy, or a combination thereof. We describe the case of a 57-year-old man who presented with a painless left testicular mass and painful gynecomastia. Serum tumor markers (alpha fetoprotein, human chorionic gonadotropin, and lactate dehydrogenase) and computed tomography of the chest and abdomen were negative. The patient underwent left radical orchiectomy. Immunohistochemical staining was consistent with a testicular GCT. He underwent a left-template laparoscopic RPLND which revealed 2/19 positive lymph nodes. Final pathological stage was IIA. He remains free of disease 32 months after surgery.

## 1. Introduction

Granulosa cell tumors (GCTs) of the testis are sex cord-stromal tumors that are represented by both juvenile and adult subtypes. Juvenile testicular GCTs account for only 1–4% of prepubertal testicular tumors but are the most common testicular stromal tumors in boys < 6 months of age [1]. Compared to the juvenile type, adult testicular GCTs are extremely rare. Stromal testicular tumors (including Sertoli and Leydig cell tumors) account for only 4% of all testicular tumors, and GCTs are the rarest of testicular stromal tumors [2]. A literature review by Schubert et al. in 2014 found only 43 cases of adult GCT described to date, and six additional reports have been made since [3–7]. Most cases of adult GCT present as a painless testicular mass. Gynecomastia may be present in approximately 17% of cases and is attributable to the tumor's synthesis of estradiol [8–10]. While most adult GCTs are benign, those that present with distant metastases confer a poor prognosis. Consensus on the optimal approach to such cases is lacking. Previous authors have proposed retroperitoneal lymph node dissection (RPLND), radiation, chemotherapy, or a combination thereof.

## 2. Case Presentation

A 57-year-old male presented for evaluation of a painless left testicular mass. He discovered the mass on self-exam. The mass had been slowly increasing in size over the previous three months. He also noted painful bilateral gynecomastia. Review of systems was negative for fevers, chills, night sweats, nausea, vomiting, fatigue, anorexia, and shortness of breath. He noticed no change in libido or erectile function. He was not aware of any palpable lymph nodes. His past medical history otherwise included atrial fibrillation and obstructive sleep apnea. He had no history of abdominal or pelvic surgery. He denied history of cryptorchidism. Family history was notable for breast and lung cancer, but no urologic malignancy.

On exam, the patient had a painless nodular mass in his left testis and a left-sided hydrocele. The right testis was unremarkable. His epididymides were normal bilaterally with no masses, tenderness, or induration. He had no palpable inguinal lymph nodes. His abdominal exam and the reminder of his genital exam were normal. Gynecomastia was present, and the breast tissue was tender to palpation.

**Figure 1:**
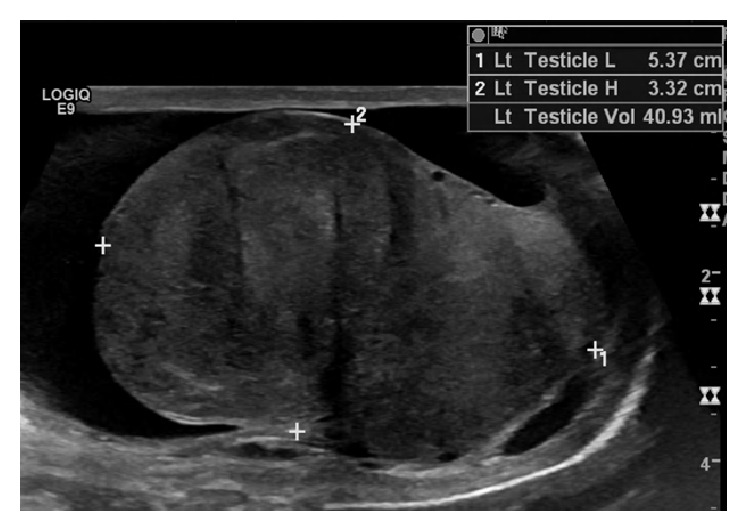
Testicular ultrasound. Longitudinal examination of the left testis demonstrates a heterogeneous mass, which has nearly replaced all normal testicular parenchyma. A small reactive hydrocele is present.

Serum blood counts and chemistries were normal. Alpha fetoprotein (AFP), human chorionic gonadotropin (hCG), and lactate dehydrogenase (LDH) were normal (0.8 ng/mL, <2 mIU/mL, and 186 U/L, resp.). A scrotal ultrasound showed a 4.5 cm hyperemic left testicular mass infiltrating and essentially replacing the left testicle, as well as a reactive left hydrocele (Figure 1). Computerized tomography (CT) scan of the chest, abdomen, and pelvis showed no retroperitoneal or pelvic lymphadenopathy and no evidence of metastatic disease.

**Figure 2:**
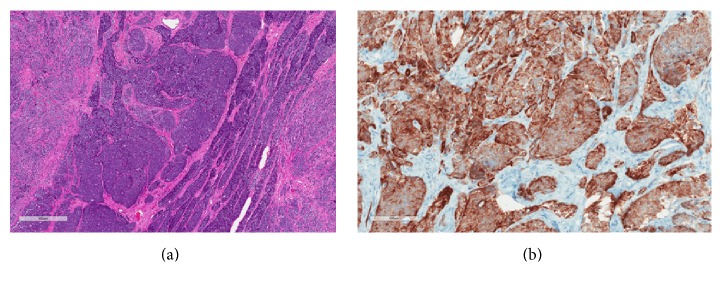
Testicular granulosa cell tumor histology. Sections of the testis show a nested neoplasm composed of undifferentiated to poorly differentiated cells infiltrating through the interstitium. The cells have scant cytoplasm and round-to-oval nuclei with occasional longitudinal nuclear groves. There is a suggestion of rosette formation in some areas, which could represent Call-Exner bodies. Mitotic figures are identified. The overall morphology is consistent with a granulosa cell tumor, adult type (a). Immunohistochemistry shows that the tumor cells are diffusely positive for inhibin (b).

The patient underwent left radical inguinal orchiectomy without complications. The left testis mass was 4.7 cm × 2.8 cm × 2.3 cm on gross examination. Histological examination revealed abundant neoplastic cells with scant cytoplasm and round-to-oval nuclei with longitudinal grooves. They infiltrated through the interstitium as nests, cords, and single cells, with focal rosetting, reminiscent of Call-Exner bodies. The immunohistochemical profile (coexpression of vimentin, inhibin, scattered cytokeratin, and CD56) was consistent with testicular GCT (Figure 2). Neuroendocrine tumor and malignant melanoma were excluded (negative chromogranin and synaptophysin; negative HMB-45 and Melan-A). Tumor invaded into the adnexa and lymphovascular space.

**Figure 3:**
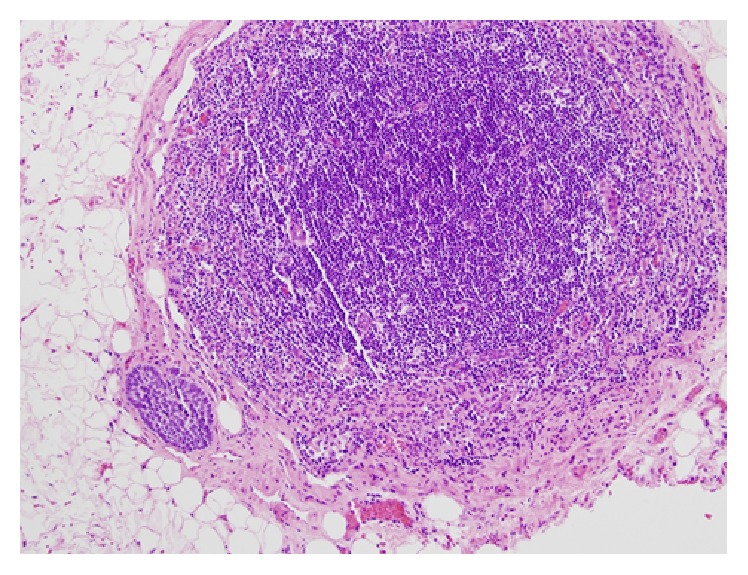
Metastatic granulosa cell tumor in an interaortocaval lymph node. A tumor nest is present in the subcapsular space of an interaortocaval lymph node. The neoplastic cells have scant cytoplasm and oval nuclei with conspicuous longitudinal groves, consistent with the patient's known primary testicular granulosa cell tumor.

The patient was counseled regarding the paucity of literature related to the management of GCT. Treatment options including RPLND, chemotherapy, and active surveillance were discussed. The patient elected to undergo left-template laparoscopic RPLND. Twelve interaortocaval lymph nodes and 7 periaortic lymph nodes were removed. Microscopic examination revealed 2/12 interaortocaval nodes with histology consistent with GCT metastases (both <2 cm in size) (Figure 3). The patient's final stage was IIA (pT2/N1/M0) GCT of the testis.

Four months after his initial presentation, the patient complained of fatigue and decreased muscle mass. He was found to have decreased serum free and total testosterone (26.5 pg/mL and 234 ng/dL, resp.), increased follicle-stimulating hormone (FSH) (53.6 mIU/mL), and increased luteinizing hormone (LH) (13.3 mIU/mL). Estradiol (18 pg/mL), thyroid-stimulating hormone (2.12 mIU/L), and free thyroxine (T4) (1.3 ng/dL) were normal. These results were consistent with primary hypogonadism. He declined testosterone replacement therapy. In the months following RPLND, he complained of worsening bilateral painful gynecomastia. He underwent bilateral mastectomy. Thirteen months after initial presentation, his free and total testosterone levels had normalized (42.8 pg/mL and 266 ng/dL, resp.) with persistently elevated FSH and LH, indicative of compensated primary hypogonadism. Blood work drawn at 26 months showed stable levels of testosterone, FSH, and LH. Surveillance CT scans of his chest, abdomen, and pelvis obtained every 6 months following RPLND have been negative for recurrence. His most recent follow-up was 32 months after initial presentation.

## 3. Discussion

Approximately 25% of adult testicular GCTs display malignant features, thus necessitating careful clinical and histopathologic investigations to evaluate metastasis and metastatic potential. However, due to the scant number of cases, factors predictive of malignancy have not yet been clearly defined. In 2011, Hanson and Ambaye reviewed all prior cases of adult GCTs (*n* = 29, with 6 being malignant) [11]. On univariate analysis, tumor size greater than 5.0 cm was the only feature statistically associated with malignancy; patient age, mitotic activity, tumor necrosis, and the presence of gynecomastia did not predict benign versus malignant behavior.

The prognosis for patients with malignant GCTs is variable and not well-defined, again owing to its infrequency. Following a review of the literature, Hammerich et al. found that patients with distant or multiple metastases (*n* = 3) progressed quickly and had limited survival (as little as five months following diagnosis). Conversely, patients with metastasis to retroperitoneal lymph nodes only (*n* = 2) tended to have a longer survival period (one patient was alive with disease at 14 months following RPLND and chemotherapy; the second patient was without evidence of disease at 168 months following RPLND and radiation) [12]. The patient presented herein is presently without evidence of disease despite lymph node positivity discovered on RPLND. Clearly, larger numbers of patients with longer follow-up periods are needed to provide more definitive prognoses for patients with metastatic spread.

To date, there are no well-established treatment guidelines for the malignant variants of adult testicular GCTs. The rarity of the condition precludes definitive management algorithms. A review of reported cases by Hammerich et al. suggests that various options may be viable and that a combination of treatment modalities (RPLND, chemotherapy, and radiation) may prove to be advantageous. Chemotherapy regimens have included bleomycin, etoposide, and cisplatin, or doxorubicin and cisplatin. In the absence of metastatic disease on cross-sectional imaging, active surveillance is also a reasonable option. It has previously been suggested, based on experience with sex cord-stromal tumors (not specifically GCTs), that RPLND may be beneficial in stage 1 disease, but less effective in N1 cases [2]. The patient in the present case has been counseled regarding the possible benefit of adjuvant chemotherapy but has elected for close active surveillance at this time.

## 4. Conclusion

The experience presented herein suggests that primary RPLND with or without neoadjuvant or adjuvant chemotherapy may be a viable option in select patients with testicular GCT. Patients may experience the sequelae of changes in testicular endocrine function and should be monitored based on symptomatology.
